# Handwriting as a Biomarker for Early Detection of Parkinson’s and Alzheimer’s Diseases: A Comprehensive Guide for Researchers

**DOI:** 10.3390/s26134190

**Published:** 2026-07-02

**Authors:** Ameur Bensefia, Nafeth Al Hashlamoun

**Affiliations:** Computer & Information Sciences Division, Higher Colleges of Technology, Abu Dhabi 41012, United Arab Emirates; nhamdi@hct.ac.ae

**Keywords:** Parkinson’s disease, Alzheimer’s disease, computer vision, deep learning, machine learning, handwriting analysis, neurodegenerative diseases, pattern recognition, biomarkers

## Abstract

Neurodegenerative diseases such as Parkinson’s disease (PD) and Alzheimer’s disease (AD) present a significant and growing challenge to the healthcare systems worldwide. Both conditions are progressive and often undetected early, making timely diagnosis crucial. Recently, breakthroughs in computer vision and artificial intelligence have enabled the development of non-invasive and cost-effective screening and decision-making tools, allowing for earlier detection of the disease. This review serves as a comprehensive guide, providing structured insights into computational research methods for automated detection of PD and AD, with focus on handwriting analysis as a subtle behavioral biomarker of neurological impairment. A range of methodologies is examined, including static and dynamic handwriting assessment, feature engineering procedures, deep learning and classical ML-based approaches. The analysis emphasizes the most effective methods, the handwriting features found to be most revealing, the datasets most used in the literature, and the performance levels reported for each disease. Several studies report that transfer learning based on convolutional neural networks and transformer-based architectures for Parkinson’s disease diagnosis is regularly able to achieve high accuracy, frequently above 95% on benchmark datasets. In contrast, Alzheimer’s disease research is progressively benefitting from multimodal approaches combining kinematic and spatial handwriting features to capture cognitive and motor changes. Structured summaries of publicly available handwriting datasets are provided, and critical advancements, ongoing challenges, and future research priorities are discussed. The integration of insights across the studies, through this work, aims to assist researchers and clinicians in the development and translation of handwriting-based, AI-guided diagnostic tools for neurodegenerative diseases.

## 1. Introduction

Neurodegenerative diseases (NDD) are a diverse set of conditions that involve progressive deterioration of neuronal structure and function. This deterioration leads to cognitive decline, motor impairment, and eventually, significant disability [1]. Among these conditions, Parkinson’s disease (PD) and Alzheimer’s disease (AD) are the two most devastating neurological diseases that have a profound impact on populations worldwide. According to the World Health Organization (WHO), about 55 million people around the world are living with dementia [2], with AD accounting for 60 to 70 percent of these cases, and PD affecting roughly 10 million people. These facts highlight the need for accessible and accurate methods to diagnose these diseases as early as possible.

Traditionally, diagnosing NDD mainly relies on clinical examination, neuroimaging modalities such as MRI and PET scans, analysis of cerebrospinal fluid, and standardized neuropsychological evaluations [3]. While these diagnostic methods can provide valuable insights, they also have major limitations. Many are expensive, not widely available in low-resource settings, or require invasive procedures. Most importantly, these tools often detect the disease at a late stage after significant and often irreversible neuronal damage has already occurred, resulting in a lost opportunity for early and more effective treatment [4].

In the last ten years, artificial intelligence (AI) technologies have revolutionized the data-driven approach to countless studies. Deep learning-based architectures, such as Convolutional Neural Networks (CNNs), have achieved models that perform similarly or even surpass human abilities in image recognition tasks [5]. Transfer learning has also become popular in medicine, as it allows pre-trained models to be adapted to new, smaller datasets in situations where clinical data may be limited [6].

These methodological advancements have accelerated the growth of research using artificial intelligence to diagnose NDD. Automated handwriting analysis can identify subtle patterns that may be missed by the human eye alone, helping to distinguish individuals affected by the disease with high accuracy. Beyond clinical screening, handwriting has long been investigated within the pattern recognition and biometrics communities as a structured neuromotor signal, where grapheme-level modeling has demonstrated the capacity to capture writer-specific motor characteristics and invariant handwriting traits [7]. Such foundational handwriting recognition and writer verification research provides important methodological grounding for current clinical applications. Furthermore, the analysis of handwriting combined with relevant behavioral modalities such as speech and gait has been proven to enhance diagnostic performance, providing clearer images of neurological function [8].

Handwriting is a highly integrated neurocognitive activity, involving motor, sensory, and cognitive systems including the motor cortex, basal ganglia and cerebellum. As a result, even transient and mild abnormalities in these systems can show up as alterations in a person’s writing. Notably, the motor systems linked to handwriting-related disorders in PD exhibit changes such as micrographia, reduced writing speed, tremor, and pen pressure, which align with motor dysfunction. Changes associated with AD are more closely linked to cognitive decline, which frequently appears as spatial disorganization, difficulty in forming letters in an optimal alignment, and alterations in letter formation and spacing [9,10].

Handwriting analysis has several advantages as a diagnostic and screening technique. It is fundamentally non-invasive and can be carried out using a pen and paper alone or through digital devices like tablets and smart pens. Handwriting also provides insights into spatial and temporal motor behaviors, generating a wealth of multidimensional information. The compact and flexible nature of handwriting-based assessment enables large-scale screening in various clinical and non-academic settings. Furthermore, the integration of digital technology and advanced computational analysis, such as pattern recognition and machine learning, makes it possible to further develop the diagnostic value of handwriting.

### 1.1. Scope and Objectives

This review analyzes handwriting-based methods for detecting the major neurodegenerative conditions, Parkinson’s disease and Alzheimer’s disease. It is envisioned as a comprehensive, one-stop guide for researchers, synthesizing the existing body of knowledge into a single rich resource. The aim is to provide structured insights and practical strategies for advancing the field, and to serve as a go-to reference for those developing, evaluating, or implementing handwriting-based AI diagnostics. The specific goals are to:

1. Review primary computational techniques used for each NDD, including both static spatial and dynamic temporal feature extraction methods.
2. Compare the performance of different methods and how researchers evaluate these approaches for each disease.
3. Classify and describe the datasets commonly used in the research community, thereby serving as an invaluable resource for researchers.
4. Evaluate current research in depth, highlighting limitations, issues, and gaps that remain in the field.

This review solely focuses on Parkinson’s disease (PD) and Alzheimer’s disease (AD), with exclusive attention to handwriting-based diagnostic tools. The primary objective is to investigate the potential of handwriting as a sensitive and informative biomarker for PD and AD, as research continues to reveal its connection to motor and cognitive impairments. Consequently, other neurologic diseases and non-handwriting-based computer vision applications are not within the scope of this work.

### 1.2. Significance and Impact

The potential of utilizing reliable, accessible and automated diagnostic tools for neurodegenerative diseases has significant implications for patient care and the health care system. Timely detection of disease can result in early therapeutic intervention, which can slow disease progression, preserve functional independence, and ultimately improve survival rate. Technology-assisted screening also offers the potential for large-scale population-wide analysis and the identification of at-risk individuals for further clinical evaluation. Quantitative and objective assessment methods are also utilized in clinical research that employs a clinical approach; they provide sensitive and reproducible outcome measures used to assess therapeutic efficacy. This review seeks to provide a unified perspective for researchers and clinicians as they explore the development of handwriting-based diagnostic methods for NDD through the consolidation of evidence from various fields.

The rest of the paper is organized as follows: Section 2 presents the clinical and biological context of NDD and the rationale for computer vision-based diagnostic approaches. Section 3 and Section 4 analyze handwriting-based detection approaches for Parkinson’s and Alzheimer’s disease, including computational strategies, feature representations, datasets, performance results, and interpretation. Section 5 discusses the results of research across both diseases, shedding light on shared challenges and methodologies. Section 6 concludes by summarizing the key findings and outlining the future directions for the integration of handwriting-based diagnostic tools in clinical settings.

## 2. Review Methodology

This work is presented as a narrative review with comparative elements, aimed at synthesizing current research on handwriting-based AI methods for diagnosing Parkinson’s disease and Alzheimer’s disease. Although not a systematic review, we followed a transparent and consistent process in selecting the literature.

### 2.1. Databases Searched

The relevant literature was identified through searches in PubMed, IEEE Xplore, Scopus, and Web of Science, and is relevant to handwriting analysis and neurodegenerative diseases.

### 2.2. Time Window

Studies published between 2010 and 2025 were included, with earlier foundational work added when necessary (e.g., key studies on handwriting biomarkers or feature engineering).

### 2.3. Search Keywords

Keyword combinations included:

○ “Handwriting analysis”, “digital handwriting”, “dynamic handwriting”,
○ “Parkinson’s disease handwriting”, “Alzheimer’s disease handwriting”,
○ “Handwriting biomarker”, “neurological handwriting features”,
○ “Machine learning handwriting”, “deep learning handwriting”,
○ “Static handwriting analysis”, “kinematic handwriting”,
○ “Digital biomarker PD”, “digital biomarker AD”.

Boolean operators (AND/OR) were applied to combine clinical and computational terms.

### 2.4. Inclusion Criteria

Studies were included if they:

○ Focused on handwriting-based assessment of PD or AD using either static images or dynamic digital signals.
○ Employed machine learning or deep learning techniques or conducted quantitative clinical/kinematic analyses.
○ Reported performance metrics, methodological descriptions, or feature sets relevant to Table 1, Table 2 and Table 3.
○ Used public datasets (e.g., DARWIN, PaHaW, UCI, HandP), institutional datasets, or well-documented proprietary collections.
○ Offered insights into feature extraction, classification, dataset characteristics, or methodological limitations.

### 2.5. Exclusion Criteria

Studies were excluded when they:

○ Focused solely on calligraphy, handwriting education, or non-clinical handwriting variation.
○ Lacked quantitative methods or handwriting data.
○ Did not provide sufficient methodological detail to support comparison.

### 2.6. Selection of Studies for Table 1, Table 2 and Table 3

Tables highlight representative and influential studies selected for their methodological relevance, dataset usage, and contribution to handwriting-based diagnostic research. These tables provide curated examples rather than an exhaustive list, illustrating key trends in methods, features, and performance across PD and AD.

### 2.7. Narrative Review Framing

Because of substantial variation across datasets, tasks, metrics, and protocols, a narrative review format was chosen. This approach allows meaningful comparison of methods while acknowledging cross-study heterogeneity and avoiding the false precision of a formal meta-analysis.

## 3. Neurodegenerative Diseases and Handwriting-Based Diagnosis

Neurodegenerative diseases are a group of disorders characterized by the progressive structural deterioration of the nervous system and its function [2]. They occur more frequently together and have common pathological features with protein misfolding and aggregation, oxidative stress, mitochondrial dysfunction and neuroinflammation [11]. However, each disease is distinguished by its specific pathological presentation, the brain regions it affects, and its course of progression.

Parkinson’s Disease is predominantly characterized by the progressive loss of dopaminergic neurons in the substantia nigra pars compacta, which results in dopamine depletion in the basal ganglia [12]. This neurochemical change results in the cardinal motor symptoms of PD: bradykinesia, tremor, rigidity, and postural instability. Non-motor symptoms, such as cognitive dysfunction, depression, and sleep disturbances are common as well [13]. Due to the insidious, progressive onset and gradual progression of PD, symptoms of dementia often result in diagnostic delays, with estimates that between 50 and 70% of dopaminergic neurons die after preclinical stages. It is believed that motor symptoms become apparent to clinical diagnosis within 7–10 years [12].

Alzheimer’s disease typically presents with gradual memory loss, cognitive impairment, difficulties with language, executive functioning issues, or visual–spatial disturbances, and cognitive decline in terms of executive function [14]. The slow buildup of amyloid-beta plaques and neurofibrillary tangles composed of hyperphosphorylated tau-protein causes widespread neuronal death, especially affecting neurons in the hippocampus and cerebral cortex [15]. In addition, the preclinical phase of AD can begin 10–20 years prior to its clinical manifestation, which makes early intervention possible if reliable biomarkers are available [16].

### Handwriting Analysis: A Horizon into Neurological Performance

Traditional clinical diagnostic approaches for neurodegenerative disorders encounter several significant shortcomings [17]. In fact, clinical diagnosis is largely reliant on the subjective evaluation of symptoms and signs, which incurs inter-rater variability and risks clinical errors. Research reports that up to 25% of PD diagnoses performed by general neurologists are not accurate [18]. In addition, by the time the clinical signs are detected, and a diagnosis is made, significant and likely irreversible neuronal damage has usually occurred. The delay in diagnosis constrains the effectiveness of current treatment and limits the ability to intervene during what should be a potentially more treatable early period of the disease. Moreover, highly developed diagnostic modalities like DaTscan imaging for PD and amyloid PET imaging for AD are costly, need advanced machinery and expertise, and are typically unavailable in low- and middle-income countries [19,20]. Finally, standard clinical investigations may not be sensitive enough to uncover relatively subtle early signs of disease, demanding the development of additional, more sensitive biomarkers.

Computer vision and AI approaches can address the majority of the challenges related to neurological disease diagnosis. Automated analysis minimizes dependence on clinical judgment by generating reproducible, quantitative measurements. These measurements can be collected over several years and across different individuals. The gathered data can then be systematically compared using automated methods, thereby decreasing the need for subjective clinical judgment. Machine learning tools also improve diagnostic accuracy by recognizing subtle trends and features that may not be visible to humans. These tools help detect disease progression with higher sensitivity and enable earlier detection. The widespread adoption of digital devices such as tablets and smartphones allows for low-cost screening in both clinical and non-clinical settings. AI systems can also combine multiple modalities, such as writing, conversation, gait, and facial expressions, to perform comprehensive evaluations that cater to various cognitive properties. Additionally, digital biomarkers aid in longitudinal monitoring of disease evolution and response, providing a long-term perspective for improved tracking of patient progress and therapeutic outcomes with high temporal resolution. This information can add value in personalized patient care and treatment outcome.

The production of handwriting comprises a series of neural pathways. Writing is a planned and initiated process that engages the prefrontal and premotor regions of the brain. The primary motor cortex, basal ganglia, and cerebellum are essential in providing the necessary synapses for fine motor control as well as for the smooth movement pathways in the brain. These regions play a pivotal role in motor processing and its coordination [21]. Visual feedback analysis and spatial organization are dependent on two regions of the brain, the parietal and occipital lobes, to recruit them. This distributed neural network means that dysfunction in any of these brain regions can present itself as changes in the appearance of handwriting traits.

In PD, the loss of dopamine in the basal ganglia circuits directly blocks the commencement, execution and amplitude of motor actions, resulting in the characteristic micrographia and bradykinetic handwriting [12]. Cognitive impairments in AD that target spatial processing, executive function, and motor planning lead to disorganized writing, spatial errors, and simplified letter forms [9]. Handwriting’s sensitivity to these neural pathways, as well as the prevalence of these conditions with the ease of data collection, positions handwriting as an ideal focus for the development of an AI model for handwriting analysis.

## 4. Parkinson’s Disease Detection Through Handwriting Analysis

Numerous investigations on automated diagnosis of Parkinson’s disease have prioritized handwriting analysis as a key approach. PD’s motor symptoms, such as micrographia and tremor, lead to clear handwriting alterations that can be easily detected by machine learning algorithms. Automated PD detection based on handwriting analysis can be classified into static and dynamic approaches. Static methods study images of finished handwriting samples, obtaining spatial and morphological features. Dynamic methods collect real-time data during the writing process on digital devices, recording both time-related and kinematic data. Recently, hybrid approaches that combine static and dynamic data have also been explored, seeking to combine the advantages of both methods for improved PD detection.

### 4.1. Feature Extraction for Parkinson’s Disease Detection

Handwriting data provides a rich source of features for classification models in PD detection. Identifying which features are most informative is essential for accurate diagnosis. As explained in the following subsections, these features can be static, dynamic, or deep learning-based, each capturing different handwriting qualities relevant to PD detection

#### 4.1.1. Static Features

A comprehensive set of spatial, shape and texture features can be derived from handwriting samples to characterize patterns indicative of neurological impairment:

- Spatial features: Key features include (1) micrographia measures (gradual decrease in letter height and width, quantified by means of bounding box dimensions and stroke magnitude); (2) stroke thickness and pressure maps (line width variations that could indicate tremor or pressure changes); and (3) spatial distribution (space between letters and words, line alignment, shape, and overall organization).
- Shape features: Relevant shape descriptors consist of (1) curvature and smoothness measures (derived from curl analysis of stroke trajectories, with PD patients tending to demonstrate decreased smoothness); (2) aspect ratios (height-to-width ratios of letters and strokes); (3) geometric moments (Hu moments and other moment-based shape descriptors).
- Texture features: Main features comprise (1) GLCM features (contrast, correlation, energy, and homogeneity from gray-level co-occurrence matrices); (2) frequency domain (Fourier transform coefficients capturing tremor-related oscillations); and (3) wavelet features (multi-scale decomposition to identify patterns at various frequency bands).

#### 4.1.2. Dynamic Features

To further refine the static handwriting descriptors provided earlier, dynamic kinematic and temporal features were extracted to identify the execution aspects of handwriting in real-time. These features reflect both the fine-grained neuromuscular control processes and the planning process involved in movement:

- Kinematic features: Important kinematic parameters encompass (1) velocity (mean, median, and standard deviation of pen velocity, slope profiles typically reflect reduced speed in PD); (2) acceleration (acceleration and deceleration patterns, jerky movements feature high acceleration variance); (3) jerk (rate of change in acceleration, quantifying movement smoothness).
- Temporal features: Temporal analysis addresses (1) stroke duration (taking each stroke to complete time in turn, prolonged in PD due to bradykinesia); (2) total writing time (total number of times for the task to be completed at hand); (3) pause times (time between strokes, as well as how often they pause); (4) in-air time (the amount of time the pen is raised, echoing planning and hesitation).
- Pressure features: These features involve (1) pen pressure (mean, variance, and range of applied pressure); (2) pressure variability (increase and decrease in pressure, which may reflect tremor or firmness).
- Complexity features: Complexity is characterized by (1) the number of strokes (decomposition of the task into constituent stroke units); (2) velocity changes (number and magnitude of changes in velocity direction); (3) entropy measures (Shannon entropy and Rényi entropy reflecting irregularity in handwriting dynamics).

#### 4.1.3. Deep Learning Features

In addition to hand-crafted spatial and kinematic descriptors, the recent literature has revealed the effectiveness and importance of deep learning models in automatically learning hierarchical feature representations directly from handwriting data. These models facilitate the detection of complicated spatial and temporal patterns without explicit predefined feature selection. The following are the main categories of deep learning features:

- CNN-learned features: Convolutional neural networks extract categorized representations, including (1) low-level features, such as edge detectors and texture patterns, learned in the early convolutional layers; (2) mid-level features, like part-based representations and local shapes, captured in the intermediate layers; and (3) high-level features, such as global patterns and abstract representations, extracted in the deeper layers.
- Recurrent network features: These features include (1) temporal dependencies, in which LSTM and GRU units process all long-term dependencies in sequential data; (2) contextual representations, where each dimensional processing captures forward and backward temporal context.
- Attention-based features: Attention mechanisms focus on salient regions, where they extract which spatial locations or types of temporal segments are better suited for classification.

### 4.2. Static Analysis Approaches

Static analysis techniques treat the handwriting as an image classification problem, using computer vision techniques to extract relevant features from captured handwriting samples. Instead of analyzing how someone writes, static analysis looks at the finished handwriting, such as a scanned page or signature.

#### 4.2.1. Traditional Machine Learning with Handwritten Features

In preliminary static handwriting analysis, researchers used classical machine learning classifiers for handwriting recognition and carefully engineered feature extraction pipelines. The aim was to describe handwriting structure though multiple local and global image features, such as: Histogram of Oriented Gradients (HOG) that gathers edge orientation and shape over the focal point of the image regions. Studies show that HOG features used in conjunction with Random Forest classifiers can reach accuracies of approximately 81.67% on spiral drawing datasets [22]. Scale-Invariant Feature Transform (SIFT) and Speeded-Up Robust Features (SURF) have also been considered where their key point descriptors capture the local interest points and enables robustness to different scales and rotations [23]. Larger local feature descriptors such as ORB and KAZE have also been assessed for PD handwriting classification but have performed much worse when compared with HOG [22].

Features are hand-crafted and commonly used as input to the classical machine learning classifiers such as Support Vector Machines (SVMs), Random Forests, K-Nearest Neighbors (KNN), and ensemble methods [24]. Although computationally efficient and interpretable, such methods necessitate extensive domain knowledge in feature engineering and may not fully capture the complexity of handwriting.

#### 4.2.2. Deep Learning and Transfer Learning

Deep learning has revolutionized static handwriting analysis for PD identification. Convolutional Neural Networks (CNNs) learn hierarchical feature representations directly from raw images, without the need for manual feature engineering. Since medical datasets are usually limited in size, transfer learning has become an effective and widely used approach. In this approach, networks pre-trained on large scale image datasets (for instance, ImageNet) are fine-tuned on handwriting data. This approach has proven effective in a range of landmark studies.

For example, Mitra et al. [25] fine-tuned a pre-trained ResNet-152 for PD detection by freezing the top 18 layers to preserve the learned characteristics whilst keeping the lower layers to perform handwriting characteristics. This approach provided an impressive 100% accuracy on the NewHandPD dataset with horizontal flip augmentation. However, this should be interpreted with caution, as small datasets can lead to overfitting.

Another study by Agrawal et al. [26] proposed a hybrid approach combining deep learning feature extraction and conventional machine learning classification. They first derived features from the fc8 layer by VGG16, then executed Binary Grey Wolf Optimization (BGWO) for feature selection, followed by SVM classification. This pipeline achieved 99.8% accuracy on NewHandPD, highlighting that feature optimization can improve performance.

Similarly, Wang et al. [27] presented an innovative feature extraction method using Vision Transformer (ViT) and Coordinate Attention-enhanced Swin Transformer (CAS). They combined the features of both architectures to model global and local dependencies, and performed weighted voting among the Logistic Regression, Decision Trees, and K-Nearest Neighbors classifiers. By using CycleGAN for data augmentation, this approach attained 92.68% accuracy on the combined HandPD and NewHandPD datasets.

Furthermore, Kamran et al. [28] evaluated six CNN architectures (AlexNet, GoogleNet, VGG16, VGG19, ResNet50, ResNet101) against four datasets: PaHaW, HandPD, NewHandPD, and Parkinson’s Drawing. These researchers managed to transform PaHaW signals into RGB images, and performed extensive augmentation including contrast adjustment, illumination change, and geometric transformation. AlexNet performance was particularly impressive, with an accuracy of 99.22% on the combined augmented dataset.

In a related investigation, Naz et al. [29] studied feature fusion across various CNN architectures. Features from AlexNet, GoogleNet, VGG16, VGG19, ResNet50, and ResNet101 were fused using addition, multiplication, and mean operations. The best result (99.35% accuracy) was attained by fusing fc6 layer features from AlexNet and VGG16 with illumination-based augmentation.

#### 4.2.3. Transformer-Based Architectures

Researchers have recently focused increasingly on transformer architectures, which have transformed natural language processing and are now being utilized for computer vision tasks. Transformers use self-attention mechanisms to learn long-range dependencies and have shown promise for handwriting analysis. The coordinate attention-enhanced Swin Transformer by Wang et al. [27] is an early application of this architecture to PD handwriting, achieving competitive performance. Another related work in [30] also assessed the Vision Transformers in PD diagnosis task.

### 4.3. Dynamic Handwriting Analysis Approaches

Dynamic handwriting analysis techniques capture the evolution of handwriting over time using digital pens, tablets, or touch screens equipped with sensors. These devices record time-series information such as pen position (x, y coordinates), pressure, velocity, acceleration, and pen-up/pen-down events. Dynamic features reveal characteristics of motor control in handwriting that cannot be captured in fixed images. The following sections highlight illustrative studies and techniques from each category.

#### 4.3.1. Kinematic Feature Construction and Traditional Machine Learning

Traditional handwriting dynamic analysis methods extract kinematic features from time-series data, which are then used as input for machine learning classifiers. The studies below showcase how these kinematic features are extracted and applied in machine learning-based handwriting analysis.

Valla et al. [31] developed new tremor-related features from Archimedean spiral drawing tests. They used Fisher’s score and recursive feature elimination for feature selection, then trained six classifiers using nested cross-validation. Notably, ensemble classifiers achieved accuracies of 84.33% on DraWritePD and 73.71% on PaHaW datasets, emphasizing the importance of well-trained and properly designed kinematic features.

Similarly, Lamba et al. [32] extracted 29 kinematic features from the UCI PD Spiral Drawings dataset, including stroke count, speed, rate of change in displacement and acceleration, velocity changes, and airtime. To address the class imbalance, they used SMOTE and applied genetic methods along with mutual information gain for feature selection. Their method achieved a notable 95.79% F1-score with nine selected features.

In a related study, Xu et al. [33] proposed a majority voting ensemble composed of six Random Forest models, each trained on diverse sensor signals from the NewHandPD dataset. Each signal was segmented with a channel length of *n* = 3000. Class imbalance was addressed using stratified five-fold cross-validation, which resulted in an accuracy of 89.40% through majority voting. In another study, Drotár et al. [34] broadened handwriting analysis by including entropy measures (Shannon, Rényi) and energy measures (cumulative energy, total kinetic energy), as well as standard kinematic features. They employed empirical mode decomposition (EMD) to generate intrinsic data patterns and applied SVM with radial Gaussian kernel for classification. Employing 168 features increased the accuracy to 85.6% from 76% for the feature-based model alone.

While many researchers relied on machine learning to analyze handwriting movement, others believe that deep learning provides even more effective solutions for sequential data. For instance, Diaz et al. [35] proposed a sequential model that combines 1D convolutions and Bidirectional Gated Recurrent Units (BiGRUs) to monitor handwriting dynamics. This sequential architecture learns time-related patterns directly from time-series data based on experience of Parkinsonian symptoms. The model achieved better performance on PaHaW and competitive performances on NewHandPD.

#### 4.3.2. Hybrid Static–Dynamic Approaches

Given the advantages of integrating static and dynamic data, many researchers have explored hybrid solutions. For example, Diaz et al. [36] presented “dynamically enhanced” static handwriting images that retain temporal and velocity information by plotting points with pen-up markers. This approach incorporates dynamic information within a static image, allowing image-based deep learning models to draw on temporal cues. This method outperformed both purely static and purely dynamic approaches, accomplishing an accuracy of 86.67% on PaHaW testing.

### 4.4. Performance Metrics and Comparative Analysis

Recent studies have investigated a variety of approaches for PD detection using handwriting analysis, which have shown divergent results in terms of performance evaluation. Table 1 presents a comparative overview of these approaches, summarizing methodologies, datasets, and reported performance metrics. In these works, transfer learning using pre-trained convolutional neural network architectures, mainly those using the VGG and ResNet methods, steadily yields some of the highest classification rates, in certain instances achieving an impressive accuracy of 100%.

However, model performance is highly sensitive to the choice of dataset. For instance, studies using the NewHandPD dataset have higher performances compared to those using the PaHaW dataset. This variance likely results from the varying dataset sizes, class balance, and task design in this case. Furthermore, the utilization of extensive data augmentation techniques, such as geometric transformations, illumination changes and synthetic data generation, can enhance the model’s robustness and generalizability.

Furthermore, various studies have demonstrated that integrating features from CNN architectures or integrating static handwriting images with dynamic temporal details can boost diagnostic performance. Conversely, approaches that solely rely on dynamic handwriting features have been reported to have relatively lower diagnostic accuracy (approximately 73–94%) compared to the static deep learning approaches (approximately 88–100%). This deviation may be primarily attributed to methodological advancements and differences in data availability.

Importantly, it is essential to be cautious when interpreting very high reported accuracies, as such results may be affected by overfitting, favorable train–test splits or limited external validation.

**Table 1:** Comprehensive comparison of Parkinson’s disease detection approaches.

Study	Year	Approach	Method	Dataset	Performance
[25]	2024	Static	ResNet-152 (fine-tuned)	NewHandPD	Acc: 100%
[26]	2024	Static	VGG16 + BGWO + SVM	NewHandPD	Acc: 99.8%
[29]	2023	Static	AlexNet + VGG16 fusion + SVM	HandPD, NewHandPD	Acc: 99.35%
[27]	2023	Static	ViT + CAS Transformer + Voting	HandPD, NewHandPD	Acc: 92.68% (NewHandPD), 88.92% (HandPD)
[28]	2021	Static	AlexNet (with augmentation)	PaHaW, HandPD, NewHandPD, Combined	Acc: 99.22% (combined), 98.31% (NewHandPD), 90.41% (HandPD), 62.50% (PaHaW)
[36]	2019	Hybrid	Dynamically enhanced images + CNN	PaHaW	Acc: 86.67%
[32]	2021	Dynamic	Kinematic features + GA/MI + ML	UCI Dataset	Acc: 100%, F1: 95.79%
[33]	2020	Dynamic	Ensemble Random Forest	NewHandPD	Acc: 89.40%
[31]	2022	Dynamic	Tremor features + Ensemble ML	DraWritePD, PaHaW	Acc: 84.33% (DraWritePD), 73.71% (PaHaW)
[34]	2015	Dynamic	Entropy/energy features + SVM	PaHaW	Acc: 85.6%
[22]	2020	Static	HOG + Random Forest	Spiral dataset (102 subjects)	Acc: 81.67%

Abbreviations: Acc = Accuracy; F1 = F1-score; SVM = Support Vector Machine; GA = Genetic Algorithm; MI = Mutual information; BGWO = Binary Grey Wolf Optimization; ViT = Vision Transformer; CAS = Coordinate Attention Swin Transformer; CNN = Convolutional nNeural Network

**Table 2:** Summary of Parkinson’s disease handwriting datasets.

Dataset	Participants (PD/HC)	Modality	Tasks	Public Availability	Key Features
NewHandPD	31/35	Image + Signal	Spiral, meander, circles, diadochokinetic	Contact authors	Both static and dynamic; balanced
HandPD	74/18	Image	Spiral, circle, meander	Public	Widely used; imbalanced
PaHaW	37/38	Signal + Image	8 tasks including spiral, writing	Public	Rich dynamic data; balanced
UCI	62/15	Signal + Image	SST, DST, STCP	Public (UCI ML Repo)	Detailed kinematics; imbalanced
DraWritePD	Not specified	Signal	Spiral, writing	Contact authors	Dynamic analysis
Kaggle	Not specified	Image	Wave, spiral	Public (Kaggle)	Simple benchmark

Abbreviations: HC = Healthy cControls; SST = Static Spiral Test; DST = Dynamic Spiral Test; STCP = Stability Test on a Certain Point; “Image + Signal” indicates availability of both static handwriting images and dynamic time-series recordings.

### 4.5. Datasets for Parkinson’s Disease Handwriting Analysis

Developing and validating automated PD detection systems demands high-quality and well-characterized datasets. This section provides a comprehensive overview of the most widely used benchmark datasets in the literature for handwriting-based Parkinson’s disease research.

#### 4.5.1. HandPD Dataset

The HandPD dataset [23] is a collection of handwritten samples specifically designed for PD research. It contains samples from two groups: (1) individuals diagnosed with PD (patients’ group); and (2) individuals without the disease (healthy control group that serves as a baseline for comparison). This dataset includes 18 healthy individuals (six males and 12 females), aged 19–79 years, and 74 PD patients (59 males and 15 females), aged 38–78 years.

In terms of handedness, participants in the control group were 16 right-handed and two left-handed with a mean age of 44.22 ± 16.53 years. A total of 69 patients from the patient group (69 right-handed and five left-handed participants) had a mean age of 58.75 ± 7.51 years. For generating data, subjects perform drawing circles, Archimedean spirals, and meanders on a prefilled form.

#### 4.5.2. NewHandPD Dataset

The revised HandPD dataset [37], referred to as NewHandPD, comprises 66 individuals divided into two groups: healthy and patient. The healthy group consists of 35 individuals with an average age of 44 years with a total 18 male and 17 female subjects, while the patient group involves 31 individuals (21 males and 10 females) with an average age of 58 years.

Twelve examinations were completed by each subject, and nine images and 12 signals were generated per subject. These examinations included spirals, meanders, circular movements, and diadochokinetic tasks. For the sample collection, handwritten dynamics were recorded using a smart pen, resulting in pictures of spirals (four), meanders (four), circles drawn on paper (one), and signals corresponding to all 12 examinations.

The dataset includes a total of 264 images and 792 signals, resulting in a more balanced and more comprehensive dataset compared to the original HandPD dataset.

#### 4.5.3. PaHaW Dataset

The PaHaW dataset was compiled at the Department of Neurology, Masaryk University, and St. Anne’s University Hospital of Brno, Czech Republic [38]. It comprises data from 37 PD subjects (19 men and 18 women) and 38 healthy control subjects (20 men and 18 women).

All participants were required to submit handwriting and drawing samples using a standardized template that included eight tasks, such as the Archimedean spiral drawing. All samples were captured on a tablet, with detailed recordings of pen movements, special coordinates, and applied pressure. The PaHaW dataset presents rich dynamic information and has been extensively used for kinematic feature extraction and time-series analysis.

#### 4.5.4. UCI Parkinson’s Dataset

The UCI Parkinson’s dataset was produced by the Department of Neurology located at Cerrahpasa Faculty of Medicine, Istanbul University [39]. It encompassed data from 62 individuals diagnosed with Parkinson disease and 15 healthy volunteers. The participants were requested to complete drawing tasks using standard coordination testing software.

Three types of tests were designed for drawing data with the aid of the graphics tablet:

- Static Spiral Test (SST): Participants were instructed to trace Archimedean spirals located on the tablet. This test is often applied in clinical research to measure motor activity, tremor, and diagnose PD. Throughout the test, relevant data, including the features mentioned earlier, were recorded.
- Dynamic Spiral Test (DST): The Archimedean spiral in this test, unlike the SST, flickers on and off at different intervals, encouraging patients to recall (not dismiss) that pattern from memory. Notably, many patients had difficulty keeping the pattern, leading to worse performance than the SST.
- Stability Test on a Certain Point (STCP): In this test, people were instructed to hold the digital pen steadily on a red dot in the middle of the screen for a certain amount of time without moving. The goal is to assess hand stability and tremor.

#### 4.5.5. DraWritePD Dataset

DraWritePD dataset highlights the handwriting patterns associated with tremor and motor control and has been utilized in studies like Valla et al. [31] for tremor-related feature detection. Its dynamic handwriting data makes it an excellent resource for performing kinematic analysis of pen trajectories and examining movement characteristics. However, it is worth mentioning that this dataset lacks publicly available data on participants, data acquisition protocols, and clinical characteristics, which restricts deeper assessment of its generalizability and clinical relevance.

#### 4.5.6. Kaggle Handwriting Dataset for Parkinson’s Disease

The Kaggle Handwriting Dataset is a widely available database and information system for Parkinson’s disease research. It contains a total of 204 static images, which are divided evenly between 102 wave drawings and 102 spiral drawings. The dataset consists solely of images with no dynamic or temporal data included. Due to its plain and open format, the dataset has been widely utilized as a benchmark for transfer learning and convolutional neural network experiments across several studies.

Nevertheless, the lack of comprehensive metadata on subjects, disease severity, and data collection conditions restricts its usefulness for clinical applications. As a result, its key role is mostly related to methodological assessment rather than practical or clinical implications.

#### 4.5.7. Dataset Comparison and Recommendations

Selecting the appropriate dataset is very crucial for effective handwriting-based PD research. The following recommendations highlight the suitability of datasets for different types of analysis and research objectives:

- For static image analysis: Researchers can refer to HandPD, NewHandPD, and Kaggle datasets as suitable benchmarks.
- For dynamic analysis: The PaHaW and UCI datasets offer comprehensive kinematic data.
- For multimodal research: The NewHandPD dataset allows researchers to compare static and dynamic approaches.
- For balanced datasets: The PaHaW and NewHandPD datasets provide more balanced class distributions compared to the HandPD and UCI datasets.

### 4.6. Current Status and Future Prospects in Handwriting-Based PD Detection

In the past five years, automatic diagnosis of Parkinson’s disease through handwriting analysis has greatly improved, reflecting methodological novelty and enhanced clinical significance. Many researchers have reported very high classification accuracy, especially when deep learning and transfer learning are used with pre-trained convolutional neural networks. In several benchmark estimates, reported accuracies have exceeded 95%, reaching the level, or in some cases, exceeding the performance of non-specialized clinical assessments. These results underscore the promise of handwriting-based AI as a supportive diagnostic tool for clinical diagnosis.

In addition to these performance improvements, the discipline has experienced remarkable methodological diversification. Researchers have employed a variety of approaches, including classical machine learning with handcrafted feature models, deep learning, ensemble frameworks, and hybrid systems based on combining static images with dynamic kinematic data. This diversity has led to comparative insights into the strengths and weaknesses of these different modeling paradigms, resulting in a better understanding of how handwriting signals encode Parkinsonian motor disabilities.

Crucially, advancements in feature analysis have revealed which handwriting dimensions are most discriminative for Parkinson’s disease. Research has consistently emphasized the relevance of micrographia-based measures, velocity and acceleration profiles, pressure variability, and tremor-induced oscillations, correlating computational findings with the clinical information available. Simultaneously, advances in technology have facilitated accessible testing, enabling affordable, non-invasive and scalable assessments through the use of tablet and smartphone technologies. Taken together, these developments establish handwriting analysis as a highly promising and continually advancing approach. It offers new opportunities for early detection and continuous monitoring of Parkinson’s disease progression.

#### 4.6.1. Limitations and Challenges

Despite the major progress, several limitations specific to PD must be addressed before considering the handwriting a reliable biomarker. Beyond the general dataset constraints and evaluation issues that are shared with AD and summarized in Section 6, PD studies face several challenges tied to the disease’s motor nature.

First, handwriting alterations in PD are heavily influenced by motor symptom profiles and medication state. Many existing datasets include only patients in stable state, or do not clearly report medication timing. This makes it difficult to know whether a model captures stable disease traits or short-term medication effects. Similarly, most cohorts are enriched for moderate to advanced PD, leading to limited evidence of model’s generalization.

Second, handwriting in PD reflects heterogeneous motor phenotypes. Tremor-dominant, akinetic-rigid, and postural-instability–gait-disorder subtypes may show distinct handwriting patterns. Current datasets are often too small or poorly annotated to analyze these subgroups separately. As a result, models learn more tremor-related patterns or micrographia, thus underrepresenting other motor dimensions.

Third, few studies explicitly address differential diagnosis within movement disorders (PD stage: multi-classification). All experiments rely on binary PD-versus-healthy control (binary classification). This setting tends to overestimate performance compared with real clinical scenarios, where the relevant differential includes atypical Parkinsonian syndromes or essential tremor. More multi-class and clinically realistic designs are needed.

Finally, the range of handwriting tasks used in PD research is still relatively narrow. Spiral drawing and repetitive tracing tasks are highly sensitive to tremor and bradykinesia but do not fully capture other aspects of motor control, such as axial symptoms or complex bimanual coordination. A broader set of ecologically valid tasks, including functional writing, such as sentence writing, signatures, or combined tasks, would give a more complete view of Parkinsonian motor dysfunction.

#### 4.6.2. Future Directions

To advance handwriting-based detection of PD towards widespread clinical adoption, several key research priorities must be addressed.

- Large-scale, multi-center studies: Future research should focus on conducting large-scale, multi-center studies to capture diverse and representative datasets making use of standardized protocols. This will facilitate the development of accurate and generalizable models.
- Longitudinal tracking: It is also important to undertake longitudinal studies to track the development of individuals over time. These studies enable assessment of disease progression as well as treatment response. Additionally, they help evaluate model predictions regarding the progression from a prodromal to a clinically manifest stage.
- Improving differential diagnosis: Another research priority is the development of models that can accurately distinguish Parkinson’s disease from related Parkinsonian syndromes such as multiple system atrophy, progressive supranuclear palsy, and corticobasal degeneration.
- Integration of explainable AI techniques: Incorporating explainable AI techniques such as attention visualization, saliency mapping, and feature attribution, is crucial. This ensures that model predictions are clinically relevant and interpretable for clinicians.
- Multimodal analysis: Researchers should also focus on multimodal analysis, integrating handwriting with complementary signals such as speech, gait, facial expressions, or olfactory measures. This type of analysis enhances diagnostic sensitivity and robustness.
- Real-world validation: Prospective clinical trials should be conducted to test diagnostic efficacy, usability, cost-effectiveness, and patient outcomes in non-controlled experimental settings.
- Standardization of protocols: Standardized protocols for data collection, preprocessing, feature extraction, and evaluation should be implemented to maintain reproducibility and enable fair comparison across studies.

## 5. Alzheimer’s Disease Detection Through Handwriting and Cognitive Assessments

Alzheimer’s disease, one of the most common types of dementia, has been extensively explored using computer vision and AI-based techniques to better understand its pathogenesis. Although the use of handwriting analysis in Alzheimer’s disease has received far less focus than in Parkinson’s disease, recent studies indicate that writing and drawing tasks can demonstrate subtle cognitive and motor alterations associated with Alzheimer’s disease pathology. This section discusses the current state of automated AD detection, particularly concerning handwriting-based biomarkers.

### 5.1. Overview of Approaches

AD detection studies employ different methodologies, reflecting the heterogeneity of cognitive decline in this condition. Unlike PD, where motor symptoms are directly expressed in handwriting kinematics, AD impacts handwriting through a heterogeneous interaction between cognitive dysfunction (executive function, spatial processing, memory) and secondary motor control impairments.

#### 5.1.1. Handwriting Dynamics and Cognitive Markers

A. *Kinematic Feature Engineering and Traditional Machine Learning*

Multiple researchers have designed feature extraction pipelines to capture cognitive and motor features of AD in handwriting. The DARWIN (Diagnosis AlzheimeR WIth haNdwriting) protocol is the most systematized approach in this area [40]. DARWIN adopts a standardized 25-task battery, including drawing geometric shapes, copying, dictated writing, and spontaneous recall. For each task, researchers extract around 18 kinematic and temporal features, resulting in around 450 derived features for the entire protocol. These features include velocity and acceleration profiles, stroke durations and pause times, pressure variations, spatial organization metrics, task completion times, and the number of strokes and pen-up events.

Several studies have applied machine learning classifiers to features derived from the DARWIN protocol for Alzheimer’s disease detection. Saha et al. [41] proposed a novel non-invasive handwriting-based framework for early Alzheimer’s diagnosis using the DARWIN dataset, which includes 174 participants. Their approach employed a stacked ensemble architecture combining Logistic Regression, XGBoost, LightGBM, and CatBoost as base learners, with a Random Forest meta-classifier to optimize predictive performance. By leveraging task-specific feature representations and ensemble learning, their model achieved approximately 97% classification accuracy. This task-aware modeling strategy recognizes that different handwriting tasks within the DARWIN battery capture distinct cognitive and motor components affected by Alzheimer’s disease, thereby improving robustness and generalization.

In a related study, Cilia et al. [42] evaluated multiple classifiers (Random Forest, K-Nearest Neighbors, Linear Discriminant Analysis) on DARWIN features. They reported approximately 91% accuracy using ensemble strategies. Ensemble approaches tend to consistently outperform single classifiers, as their work shows.

B. *Feature Selection and Gradient Boosting Methods*

A task-optimized approach utilizing forward–backward selection with XGBoost showed that a small subset of carefully selected tasks and features could approach full-protocol performance [43]. This report has shown XGBoost’s AUC of 91.37% and accuracy of 91.32%, with ensemble configurations achieving up to 97% accuracy. Reducing assessment burden without compromising predictive accuracy is particularly important for clinical deployment. Similarly, recent ensemble-based approaches applied to the DARWIN dataset have shown that stacking and hybrid voting strategies can achieve high diagnostic performance (≈95–97% accuracy) while limiting the number of required tasks, reinforcing the feasibility of task-aware and ensemble learning strategies for practical Alzheimer’s screening [44].

#### 5.1.2. Representation Learning and Automated Feature Engineering

In addition to feature engineering, some studies explore methods for automatically identifying informative representations from raw data. For instance, Azzali et al. [45] employed vectorial genetic programming to automatically extract features from raw handwriting time-series, encompassing x and y coordinates as well as pressure measurements. VE-GP evolves lists of candidate feature transformations and selects combinations that yield the highest classification performance. This method eliminates the need for manual feature composition, resulting in compact and interpretable models. It also achieves competitive accuracy with hand-engineered features and has been found to provide complementary insights beyond what regular methods can offer.

Building on automated representation learning, several studies have explored transforming raw handwriting time-series into image-based representations suitable for convolutional neural networks. For example, Białek et al. [46] converted online handwriting signals into multi-channel time–frequency spectrograms using sliding window techniques and trained CNN models on these representations. Their approach achieved competitive performance in distinguishing AD from controls (F1-score ≈ 89%), while also highlighting that optimal temporal window lengths may vary across diseases, suggesting disease-specific temporal dynamics in handwriting signals.

#### 5.1.3. Other Architectures

Transformers architectures have been considered for AD detection due to their proven success in natural language processing and computer vision. Recent studies have explored transformer–CNN fusion models to analyze AD based on handwriting diagnosis, achieving an accuracy of approximately 87.99% [47]. Due to self-attention mechanisms in transformers, long-range dependencies can be modeled in handwriting sequences, which may be indicative of subtle disruptions in motor planning and execution.

More recently, Gong et al. [48] proposed a multimodal Hybrid Transformer framework that jointly integrates 2D handwriting images and 1D dynamic signals from the DARWIN dataset. By incorporating a gated hybrid attention mechanism that blends similarity and difference attention across modalities, their approach captures complementary spatial and temporal patterns. This model achieved state-of-the-art performance on DARWIN, reporting an F1-score of 90.32% and accuracy of 90.91% on Task 8 (“L” writing), improving upon prior methods.

The Clock Drawing Test (CDT) is another popular cognitive screening approach that assesses executive function, visuospatial abilities, and semantic memory. Automating CDT scoring with the support of computer vision has various benefits. For example, Qian and Liao [49] developed a mobile intelligent screening application for AD that makes use of the CDT. Their system utilizes a smartphone camera to capture clock drawings and employs computer vision to retrieve features such as the number order, hand placement, and overall arrangement. Additionally, it uses AI to recreate clinical scoring criteria and supports remote screening and frequent assessment. Although these features are highly promising for accessibility, the majority of CDT automation work currently focuses on reporting preliminary results without any broader validation.

### 5.2. Feature Extraction for Alzheimer’s Disease Detection

#### 5.2.1. Handwriting Dynamic Characteristics

The dynamics of handwriting offer valuable insights into the temporal organization, motor control, and the cognitive planning involved in writing, all of which are particularly important for the identification of Alzheimer’s-related disabilities. In the context of handwriting analysis for AD, the characteristics can be grouped into three main categories:

- Temporal characteristics: Temporal analysis addresses the following: (1) Stroke time: The duration of various strokes. Individuals with AD often exhibit long stroke times, which reflects cognitive slowing. (2) Pause profiles: The duration and frequency of pauses. More frequent and longer pauses may indicate difficulties with motor planning. (3) Total writing time: The overall time elapsed to finish writing or drawing tasks. Longer total time can be a sign of cognitive or motor impairment. (4) In-air time ratio: The proportion of time the pen is raised. A longer in-air time may indicate slow planning or hesitation during writing.
- Kinematic features: Important kinematic features encompass the following: (1) Velocity patterns: The average and variability of writing velocity. In AD, decreased speed and increased variability are often noticed. (2) Acceleration: The smoothness of acceleration and deceleration. Individuals with AD normally show uneven acceleration. (3) Jerk metrics: Measurements to determine the smoothness of movement. Higher jerk values indicate less fluid movement.
- Pressure features: These features involve the following: (1) Pen pressure: The mean and variability of pressure applied. Some studies report affected pressure control in individuals with AD. (3) Pressure–velocity coupling: the relationship between pressure and velocity. This relationship may be affected by Alzheimer’s disease.

#### 5.2.2. Spatial and Cognitive Characteristics

In addition to temporal and kinematic mechanics, handwriting’s spatial organization and structure represent higher-order visuospatial and cognitive functions, which are commonly disrupted in Alzheimer’s disease. These features provide complementing indices of impairment during writing and drawing tasks. Key categories include:

- Spatial organization: Key features are (1) letter and word spacing: irregular spacing that reflects visuospatial dysfunction; (2) line alignment: difficulty maintaining horizontal writing lines; (3) margin adherence: tendency to drift from the intended writing area; and (4) size consistency: variability in letter size and proportions.
- Shape and form features: Relevant aspects include (1) letter formation quality: simplified or distorted letter shapes; (2) stroke thickness variations: alterations in line width and consistency; and (3) tremor indicators: high-frequency oscillations in handwriting trajectories.
- Task-specific cognitive markers: Important indicators comprise (1) Clock Drawing Test (CDT) features, including number placement accuracy, clock hand positioning, presence of all required elements, and overall spatial organization; and (2) copy versus recall: performance differences between copying tasks (often preserved) and recall tasks (typically impaired) can help differentiate AD.

### 5.3. Performance Metrics and Comparative Analysis

Table 3 illustrates an overall trend for handwriting-based Alzheimer’s disease detection in the literature. Protocols based on a comprehensive list of handwriting tasks, such as the DARWIN dataset with 25 handwriting tasks, have led to the most systematic studies, with multiple authors declaring that accuracy between 90 and 97% has been achieved using different machine learning methods. Across the works, gradient boosting algorithms, particularly XGBoost and its associated methods, are among the most reliable ones. They are renowned for their high prediction accuracy and intrinsic feature importance analysis, which enhances interpretability.

Crucially, multiple studies show that when balancing the selection of tasks and applying feature reduction, it is possible to achieve performance on par with full assessment protocol while significantly reducing patient and clinician burden. This result indicates that diagnostic accuracy can be maintained or even increased, while also increasing diagnostic efficiency. Furthermore, recent evidence suggests that multimodal approaches, such as combining handwriting analysis with cognitive measures (e.g., the Clock Drawing Test), may contribute to improved diagnostic effectiveness. Whereas static handwriting image analysis for Parkinson’s disease is well established in Parkinson’s disease research, Alzheimer’s disease studies have predominantly focused on dynamic handwriting signal, thus leaving static image-based methods under-researched. Additionally, the majority of studies mentioned in the present work have relatively small sample sizes, usually between 100 and 300 individuals, which could affect the generalizability of the results, emphasizing the need for larger, more heterogeneous cohorts.

**Table 3:** Comprehensive comparison of Alzheimer’s disease detection approaches.

Study	Year	Approach	Method	Dataset/Cohort	Performance
[41]	2025	Dynamic	RF, LR, SVM task-specific ensembles	DARWIN (~174 subjects)	Acc: ~97%
[42]	2019	Dynamic	RF, KNN, LDA ensemble	DARWIN	Acc: ~91%
[44]	2025	Dynamic	Ensemble-based	DARWIN	Acc: ~95–97%
[43]	2025	Dynamic	Task + feature selection + XGBoost	DARWIN-like	AUC: ~0.9137, Acc: ~0.9132
[48]	2024	Dynamic	Hybrid Transformer	DARWIN	F1: 90.32% Acc: 90.91%
[45]	2022	Dynamic	Vectorial Genetic Programming	Tablet time-series (<300)	Competitive with hand-engineered features
[46]	2024	Dynamic	CNN on spectrograms	Mixed	F1: 89.8%
[47]	2024	Dynamic	Transformer and CNN fusion	Handwriting images	Acc: ~87.99%
[49]	2021	Static	Mobile CDT scoring	Study-specific	Prototype demonstrated

Abbreviations: Acc = Accuracy; F1 = F1-score; AUC = Area Under the ROC Curve; RF = Random Forest; LR = Logistic Regression; SVM = Support Vector Machine; KNN = K-Nearest Neighbors; LDA = Linear Discriminant Analysis; CNN = Convolutional Neural Network.

### 5.4. Datasets for Alzheimer’s Disease Handwriting Analysis

#### 5.4.1. DARWIN Dataset

The DARWIN (Diagnosis AlzheimeR WIth haNdwriting) dataset is one of the most extensive and commonly used datasets for Alzheimer’s disease handwriting [40]. The cohort sizes for representative studies are around 100 to over 200 participants, including patients diagnosed with Alzheimer’s disease, healthy individuals, and sometimes participants with mild cognitive impairment.

The data are collected using a digital tablet and stylus, following a conventional 25-task protocol recording extensive x–y pen trajectories, timestamps and pressure levels. Tasks covered are cognitive, motor and geometric drawings, copying exercises, dictated writing, recall-based writing, and clock drawing, all aligned with clinically standard procedure. For each task, around 18 features are collected, yielding about 450 features in the complete protocol, comprising kinematic, temporal, pressure-related, and spatial aspects.

The dataset is publicly available and has been widely used by several research groups, allowing for reproducibility and cross-study comparison. Owing to its standardized design and sufficient task diversity, DARWIN has supported the development of task-specific, optimized or ensemble models, which are considered fundamental to handwriting-based Alzheimer’s disease research.

#### 5.4.2. Institutional and Study-Specific Datasets

Several Alzheimer’s disease handwriting studies have employed single-institutional or specific research-project databases, including:

- Spectrogram-Based Study Dataset [50]: Includes 42 healthy controls, 21 AD patients, 35 PD patients, and 15 Parkinson’s disease mimics. Multiple handwriting tasks are performed, with time-series data transformed into spectrogram representational formats.
- Offline Handwriting Image Datasets [40]: Comprise several study-specific sets containing scanned and photographed handwriting samples. Access to this dataset is restricted and not fully open to the public.
- Clock Drawing Mobile App Datasets [47]: Data in this dataset are obtained through smartphone applications, and the sample sizes are typically not described. These datasets typically concentrate on the CDT characteristics.

#### 5.4.3. Dataset Limitations and Needs

Some of the critical limitations of existing datasets used in handling handwriting-induced Alzheimer’s disease research hinder methodological thoroughness and clinical translatability. Most available datasets are from single institutions or narrow geographical regions, limiting demographic diversity and limiting the generalizability of trained models across populations. Sample sizes are usually small, ranging between 100 and 300 clients, which is not enough to train and validate deep learning models that are robust to overfitting issues. Moreover, representation across different stages of the disease remains inconsistent, with only a limited number of datasets capturing individuals in the prodromal stage (mild cognitive impairment) or featuring longitudinal data to assess cognitive decline over time. Other common comorbidities in Alzheimer’s disease (such as vascular diseases, as well as depression) are also poorly represented, which limits the ability of the models to perform reliably in clinical environments. In addition, there is a great deal of inconsistency in the design of handwriting tasks, data acquisition hardware, and the protocols used for data collection across studies, making it difficult to compare and collate results.

Overcoming these limitations will necessitate a new generation of datasets that are specifically designed for scalability, diversity, and clinical relevance. Ongoing research into multi-center data collection projects with more than one thousand participants provides an opportunity for substantial deep learning and subgroup analyses. Longitudinal data that closely monitor individuals as they transition from mild cognitive dysfunction to Alzheimer’s disease will play a crucial role in enabling early detection and understanding disease progression. In-depth demographic diversity in ethnicity, language, and educational background will be a prerequisite for fair assessment and generalizability. Furthermore, combining information on handwriting with complementary data sources (neuroimaging, biological biomarkers, neuropsychology) will enable a more comprehensive classification of disease mechanisms. The transition to standardized protocols across centers would enable cross-study pooling, meta-analysis, and replicability of results, accelerating the translation of handwriting-based AI tools into clinical practice.

### 5.5. Current Status and Future Prospects in Handwriting-Based AD Detection

The reliance on handwriting to detect Alzheimer’s disease has been extensively researched, with considerable progress made in identifying how the disease is reflected in writing behavior. Research has demonstrated that changes in handwriting patterns in Alzheimer’s disease result from a combination of factors, comprising cognitive slowing, decreased executive function, visuospatial dysfunction, and motor control loss. The well-structured DARWIN dataset has facilitated a better understanding of how Alzheimer’s disease affects handwriting, helping in the identification of the most discriminative handwriting features associated with cognition decline amongst those typically used in handwriting analysis.

Methodologically, machine learning approaches have shown notable and sustained performance. Gradient boosting techniques, particularly XGBoost, have demonstrated classification accuracies of 85% to 97% and maintained interpretability that is essential for clinical application and trust. This tradeoff between performance and transparency is especially important in cognitive testing scenarios.

A key practical achievement is the observation that carefully chosen subsets of handwriting tasks can yield results comparable to those obtained from the full test protocol. Task optimization has been demonstrated to minimize both the time required for assessment and the participant burden without any loss in diagnostic precision. Relatedly, automated scoring systems for standard cognitive tests, such as the Clock Drawing Test, have demonstrated that well-established neuropsychological tests can be successfully digitized. These systems contribute to the development of scalable, objective and extensively adopted cognitive screening tools.

#### 5.5.1. Limitations and Challenges

The detection of AD based on handwriting still faces important challenges that are partially shared with PD and partially specific to cognitive disorders. General limitations related to dataset size, class imbalance, and evaluation practices are discussed in Section 6; here we focus on aspects that are particularly critical for AD.

The first issue is the cognitive heterogeneity of AD. Indeed, patients differ in the degree to which memory, executive function, visuospatial abilities, and language are affected, especially in the early stages. As a result, a single handwriting task or feature set is unlikely to capture the full range of impairment, making consistent pattern detection difficult across individuals.

Second, some handwriting alterations observed in AD are not specific to Alzheimer’s disease. Slowed writing, increased pauses, and mild spatial disorganization can also occur in normal aging, depression, vascular disease, or other dementias. When control groups are restricted to healthy older adults, models may inadvertently learn to distinguish “impaired” from “healthy” rather than AD from other clinical conditions. Studies that include mixed dementia cohorts and psychiatric controls are still rare.

Third, handwriting is strongly influenced by education level, literacy, and premorbid writing habits. Individuals with lower educational attainment or less writing experience may show poorer baseline performance even in the absence of neurodegenerative disease. If these factors are not carefully documented and balanced across groups, there is a risk that models will use educational or cultural proxies instead of disease-related features, introducing bias and reducing generalizability.

Fourth, AD studies often depend on complex multi-task protocols (such as full DARWIN assessments), which may be burdensome for frail or advanced-stage patients. Long protocols increase missing data and limit applicability in routine care or remote settings. Although recent work on task and feature selection has shown that shorter batteries can retain high diagnostic accuracy, these optimized subsets still require broader validation across independent cohorts.

Finally, there is ongoing uncertainty about how handwriting-based indices relate to established AD biomarkers. Studies that link handwriting features to neuroimaging, cerebrospinal fluid markers, or standardized cognitive scales, are still rare. Without these convergent validations, it remains unclear whether handwriting captures AD-specific pathophysiology or more general cognitive decline. Greater integration with other biomarkers is needed to clarify its diagnostic relevance.

#### 5.5.2. Future Directions

Many of the future research directions and priorities for handwriting-based Alzheimer’s disease detection are associated with those identified for Parkinson’s disease, illustrating shared challenges and opportunities for progress in both fields. To advance the field of handwriting-based AD detection, the following directions are suggested:

- Large-scale collaborative studies: Future research should prioritize large-scale multi-center collaborations to create diverse and representative datasets using standardized formats. These strategies will further reinforce the development of effective and generalizable models for Alzheimer’s disease detection.
- Longitudinal tracking: Longitudinal research is of paramount importance, particularly in studies that track individuals from cognitively normal status to mild cognitive impairment, and eventually to Alzheimer’s disease. Such studies can help explain the disease progression over time, enable early detection, and support predictive modeling.
- Improving differential diagnosis: There is a need to design models that are able to differentiate Alzheimer’s disease from other dementias, such as frontotemporal dementia, Lewy body dementia, vascular dementia, and depression to enhance clinical relevance.
- Integration of explainable AI techniques: Artificial intelligence approaches should be incorporated to facilitate clinical implementation by explaining model predictions in a clear and clinically interpretable manner, helping to build increased clinician trust and acceptance of the model.
- Real-world validation: Prospective clinical trials should be performed to test not only diagnostic accuracy but also clinical utility, cost-effectiveness, and impact on patient outcomes in everyday care settings.
- Multimodal analysis: Researchers should focus on multimodal analysis, integrating handwriting with complementary signals such as speech, gait, facial expressions, or olfactory measures. This type of analysis enhances diagnostic sensitivity and robustness.
- Standardization of protocols: Standardized protocols for data collection, preprocessing, feature extraction, and evaluation should be implemented to maintain reproducibility and enable fair comparison across studies.
- Personalized digital phenotyping: The next generation of research should adopt digital phenotyping through smartphones and wearables to capture handwriting and its correlated behaviors within naturalistic contexts. Personalized baseline models that track changes at the within-person level rather than group-level measures over time may increase sensitivity to early cognitive deterioration and foster more personalized methods of assessment.

## 6. Synchronous Cross-Disease Synthesis and Discussion

Studying detection methods for AD and PD can emphasize the methodological similarities as well as the unique approaches adopted for each disease. Notably, both AD and PD exhibit a dominant trend, which appears to be the extensive use of transfer learning paired with pre-trained CNN. This method has particularly proven effective in compensating for limited labeled medical information by utilizing representations learned from large non-medical datasets. As a result, strong performance can be achieved even when task-specific data are not easily accessible.

Integration of multimodal data in AD and PD detection represents another major convergence. Research consistently shows that the fusion of complementary modalities, such as combining static and dynamic handwriting in Parkinson’s Disease, or integrating handwriting with speech and neurophysiological signals in Alzheimer’s Disease, is effective in identifying the full spectrum of disease-induced deficits. Therefore, this multimodal perspective is especially relevant considering that diseases are present in multiple cognitive and motor domains, and single-modality approaches may not accurately characterize their complexity.

The methodological research within both diseases emphasizes a fruitful tension between feature engineering and end-to-end deep learning. Clinical insights and handcrafted features play a vital role in improving interpretability and ensuring that model behavior is consistent with established disease mechanisms, which are fundamental for clinical adoption. Alternatively, end-to-end deep learning models have demonstrated strong performance in several datasets because they can learn the complex and high-dimensional patterns that are hard to encode manually. Hybrid techniques, which combine engineered features across deep learning pipelines, are emerging as an effective tradeoff. This strategy helps maintain transparency with predictive performance.

### 6.1. Shared Methodological Challenges Across PD and AD

Despite these advances, handwriting-based AD and PD studies share several methodological issues that limit generalizability and validity as biomarkers. Bringing these common limitations together avoids repetition in the disease-specific sections and clarifies the obstacles faced by both fields.

A first group of challenges concerns dataset constraints. Many studies rely on small cohorts, often fewer than a few hundred participants, with imbalanced distributions between patients and controls and limited demographic diversity. Most datasets are collected at single centers and do not cover the full range of disease severity or comorbidities. These factors restrict statistical power and make it difficult to train models that generalize to new populations or early disease stages.

A second group involves evaluation design and the risk of overfitting. Reported accuracies above 95–100% are common, especially on small or highly curated datasets. However, these results are often based on random sample-level splits, heterogeneous cross-validation schemes, or selective reporting of the best configuration. Participant-level data leakage, where samples from the same subject appear in both training and testing sets, can seriously inflate performance scores. In addition, heavy data augmentation, while helpful for robustness, may introduce artificial patterns that do not correspond to real clinical variability. Similar concerns regarding synthetic data generation and the need to verify distributional fidelity and explainability consistency have been reported in rare neuromotor disorder classification studies employing generative adversarial networks [51].

Third, external validation testing remains rare. Most models are developed and evaluated on the same dataset, with few studies testing their performance on independent cohorts or across different acquisition sites. The lack of multicenter and prospective validation raises questions about how well current models would perform in everyday clinical settings.

Fourth, both AD and PD studies face confounding factors related to task design, demographics, and hardware. Certain tasks may primarily measure tremor or general cognitive slowing rather than disease-specific mechanisms; age, education, language, and writing habits can affect handwriting independently of disease; and variation in tablets, sampling rates, and stylus sensors can introduce domain shifts between datasets. If these factors are not carefully controlled or reported, models may learn site-, device-, or cohort- specific signatures instead of generalizable disease markers.

Taken together, these shared challenges underline the need for standardized protocols for data collection, transparent reporting of experimental design, subject-wise validation splits, and more systematic cross-dataset evaluation across both PD and AD handwriting research.

An additional and important limitation concerns the stage of disease represented in current datasets. Most available handwriting datasets for both PD and AD consist of individuals with already confirmed clinical diagnoses, frequently in moderate or clearly symptomatic stages. Therefore, models are trained to distinguish manifest disease from healthy controls rather than to detect subtle preclinical or very early neurodegenerative changes. Although handwriting is often proposed as a tool for early screening, robust evidence demonstrating reliable detection in prodromal PD or in preclinical and mild cognitive impairment stages of AD remains limited. Longitudinal studies in at-risk populations are therefore essential to determine whether handwriting biomarkers can truly capture early neurodegeneration rather than established clinical impairment.

To consolidate these reproducibility considerations, Table 4 provides a structured synthesis of validation design, imbalance handling, calibration reporting, and code availability practices across the reviewed studies.

### 6.2. Disease-Specific Drivers, Tasks, and Endpoints

While PD and AD share common computational frameworks, the mechanisms that drive handwriting alterations and the design of effective tasks and endpoints differ substantially. Clarifying these differences helps avoid overly generic conclusions.

In Parkinson’s disease, handwriting changes are primarily driven by motor circuit dysfunction in the basal ganglia. Micrographia, tremor, reduced movement amplitude, and bradykinesia are directly reflected in stroke size, velocity, acceleration, and pressure variability. Accordingly, PD studies typically focus on drawing tasks such as spirals, meanders, and repetitive tracing, which are particularly sensitive to tremor and fine motor control. Performance endpoints often include classification of PD versus controls, estimation of motor severity, or stratification by motor subtype or medication state.

In Alzheimer’s disease, handwriting alterations arise mainly from cognitive decline, including impaired planning, memory, and visuospatial organization, with secondary motor contributions. AD protocols therefore emphasize cognitively demanding tasks such as copying and recalling complex figures, writing under dictation, and clock drawing. Dynamic features related to pauses, in-air time, task completion time, and spatial organization of writing lines are especially informative. Endpoints commonly focus on differentiating AD from cognitively normal older adults or from mild cognitive impairment, and on mapping handwriting features to cognitive test scores.

These contrasting drivers have practical implications. In PD, the key question is often whether handwriting captures subtle motor abnormalities early enough to complement classical motor scales. In AD, handwriting functions as a digital cognitive proxy, serving as a digital proxy for executive function and visuospatial abilities. As a result, task selection, feature prioritization, and clinical interpretation must be tailored to the specific disease context even when similar algorithms are used.

### 6.3. Handwriting: A Neurological Biomarker: Advantages and Disadvantages

Handwriting emerges as a powerful—though imperfect—neurological biomarker when evaluated in the context of both Alzheimer’s and Parkinson’s care. Its main advantages are its non-invasive and low expenditure characteristics, which are particularly suitable for extensive screening and repeated examinations. Handwriting is an integrated form of cognitive planning, visuospatial processing, motor execution, and sensorimotor feedback in both AD and PD. This integration allows it to capture subtle disease-relevant dysfunctions in a single naturalistic task. Its system-wide nature also enables tracking on an ongoing basis, which is particularly useful for monitoring disease progression in PD and cognitive decline in AD. In addition, handwriting information can be retrieved on digital technology in a variety of clinical and non-clinical scenarios, providing rich, multidimensional information that overcomes the limitations of basic pen-and-paper tests.

Nevertheless, these advantages are tempered by critical limitations that impact the detection of both AD and PD. One major challenge is the limited disease specificity: handwriting changes are not exclusive to either condition, and can also be a result of normal aging, other neurological disorders, or psychiatric conditions, making differential diagnosis challenging. Education level, cultural writing habits, and pre-existing motor skills can considerably affect handwriting, presenting confounding factors that might cause bias in AI models if not suitably controlled. Handwriting changes are often too subtle to be detected reliably in the early stages of AD and PD, limiting sensitivity for preclinical or prodromal diagnosis. Also, as handwriting necessitates a minimum level of motor ability, its applicability is limited in patients with advanced PD or severe motor impairment. Consequently, patients who have the most clinical need may not be included.

### 6.4. Path Toward Clinical Translation

In order to realize clinical relevance for handwriting-based, AI-based diagnostics in AD and PD, a range of key challenges must be tackled to make a meaningful impact. We need rigorous validation, which requires large, multi-center, prospective studies, including external validation cohorts of real-world clinical populations. These studies need to include suitable statistical controls and benchmark the performance of AI against known clinical gold standards and not against individual accuracy metrics.

Clinical utility must be demonstrated well beyond technical validation. For AD and PD, this means demonstrating that AI-enabled handwriting analysis improves diagnostic timing, facilitates tracking of progression of disease, reduces health spending, or aids clinical decision-making where traditional tools are ineffective. High predictive accuracy by itself is not enough without proof of real value for patients and clinicians. Another significant barrier is regulatory approval. The adoption of these applications has highlighted the need for robust implementation frameworks. Translating such tools into routine care requires adherence to regulatory pathways like FDA clearance or CE marking, both of which demand robust proof of safety, effectiveness, reproducibility and quality management.

Furthermore, the implementation of the principles of implementation science (implementation science is the study of how to best put research findings, new technologies, or evidence-based practices into real-world use—especially in healthcare and public health) is critical for effective adoption, as these principles specifically address practitioner training, adoption into clinical workflows, reimbursement models and acceptance of the device among patients. For chronic diseases like AD and PD, these are particularly critical components for effective long-term care.

Lastly, ethical dimensions are critical for responsible deployment. AI systems need to be verified for fairness in all segments of the population, especially given that handwriting can vary with education, culture, and aging. Privacy protections, informed consent as well as human supervision in AI recommendations are essential for building trust and confirming that these technologies complement, rather than replace, clinical judgment.

## 7. Conclusions

This review summarizes the current state of research on handwriting-based AI diagnostics for Parkinson’s and Alzheimer’s diseases, drawing attention to both their promise and the unresolved issues in this field. Handwriting, being both a non-invasive and cost-effective biomarker, captures the subtle interactions between cognitive and motor functions affected by neurological disorders. Handwriting-based diagnostics for PD have shown impressive accuracy, particularly when utilizing transfer learning and deep learning for both static and dynamic data. The integration of multiple feature types in hybrid models provides additional performance improvements. Handwriting-based analysis in Alzheimer’s disease, particularly using structured methods like DARWIN, has also shown promising results for cognitive screening and automated test scoring. When combined with machine learning and deep learning techniques, these approaches achieve very good performance; however, development in this field continues to lag behind progress made in PD. This is due to smaller datasets, a narrower range of methods, and limited clinical implementation.

Although these findings are promising, there are still major barriers for clinical translation in both diseases. Constraints inherent in the datasets, including small and demographically confined samples, class imbalance, variability in the evaluation approach and limited testing in the external or prospective environment, hinder generalizability and real-world application. Clinical adoption is also hampered by methodological features, such as overfitting and the use of black box models. Additionally, there is a lack of studies focusing on differential diagnosis, longitudinal disease development, and implementation in routine care. Furthermore, practical issues involving data entry, workflow integration, and usability have not been adequately addressed.

In the future, clinical impact will ultimately rely on collective efforts among various dimensions. Wide-scale, multi-center data collection with standard protocols are needed, along with longitudinal studies to better understand disease progression in PD and cognitive decline in AD. The introduction of handwriting with adjunctive biomarkers is an urgent next step. Developing explainable AI models that are consistent with clinical reasoning is also crucial. Furthermore, conducting prospective clinical trials to demonstrate real-world utility is very critical for progress. Tackling those barriers through interdisciplinary cooperation amongst clinicians, data scientists and implementation researchers will in the end show whether such tools could be beyond the “proof-of-concept” phase.

Nevertheless, handwriting-based AI systems represent a compelling opportunity for earlier detection. Maintaining methodological scrutiny and ensuring effective incorporation into clinical practice will be crucial for these tools to have a meaningful impact. With careful development and adoption, these systems can make a real contribution towards improving diagnosis and care for people afflicted by these forms of neurodegenerative diseases.

## Figures and Tables

**Table 4:** Summary of reproducibility and validation practices in handwriting-based PD and AD studies.

Aspect	Typical Practice	Key Limitation	Recommended Practice
Validation split strategy	Random sample-wise cross-validation or hold-out splits	High risk of participant-level data leakage when multiple samples per subject are present; optimistic performance estimates	Strict subject-wise (participant-level) splits to ensure independence between training and testing
External validation	Rarely performed	Limited evidence of generalizability across cohorts, sites, or acquisition protocols	Evaluation on independent external cohorts and, where possible, multi-center validation
Dataset size and composition	Small to moderate cohorts (typically <300 participants), often single-center	Limited statistical power; poor coverage of early or prodromal disease stages	Larger, multi-site datasets with broader demographic and clinical diversity
Class imbalance handling	Data augmentation, oversampling (e.g., SMOTE), or synthetic data generation	Potential introduction of optimistic bias and distributional distortion if applied at sample level	Imbalance handling performed at the subject level with transparent reporting of procedures
Performance reporting	Accuracy and F1-score most commonly reported	Accuracy alone may be misleading in imbalanced or small datasets	Use of complementary metrics (AUC, sensitivity, specificity) and reporting of confidence intervals
Model calibration	Rarely reported	Overconfident predictions	Calibration analysis
Code availability	Often unavailable	Limited reproducibility	**Public code and protocols**

## Data Availability

The study is based on previously published and publicly available datasets described in the referenced literature. No new patient data were collected for this work. All data preprocessing and analysis procedures are described in sufficient detail to ensure reproducibility.
